# Clinical and cytogenetic features of a Brazilian sample of patients with phenotype of oculo-auriculo-vertebral spectrum: a cross-sectional study: Características clínicas e citogenéticas de uma amostra brasileira de pacientes com fenótipo de espectro óculo-aurículo-vertebral: um estudo transversal

**DOI:** 10.1590/1516-3180.2013.7762204

**Published:** 2014-10-17

**Authors:** Alessandra Pawelec da Silva, Rafael Fabiano Machado Rosa, Patrícia Trevisan, Juliana Cavalheiro Dorneles, Camila Saporiti Mesquita, Vinicius Freitas de Mattos, Giorgio Adriano Paskulin, Paulo Ricardo Gazzola Zen

**Affiliations:** I MD. Postgraduate Student, Postgraduate Program in Pathology, Universidade Federal de Ciências da Saúde de Porto Alegre (UFCSPA), and Clinical Geneticist, Universidade Federal de Ciências da Saúde de Porto Alegre (UFCSPA) and Complexo Hospitalar Santa Casa de Porto Alegre (CHSCPA), Porto Alegre, Rio Grande do Sul, Brazil.; II PhD. Clinical Geneticist, Universidade Federal de Ciências da Saúde de Porto Alegre (UFCSPA), Complexo Hospitalar Santa Casa de Porto Alegre (CHSCPA) and Hospital Materno Infantil Presidente Vargas (HMIPV), Porto Alegre, Rio Grande do Sul, Brazil.; III MD. Postgraduate Student, Postgraduate Program in Pathology, Universidade Federal de Ciências da Saúde de Porto Alegre (UFCSPA), and Pharmacist, Pontifícia Universidade Católica do Rio Grande do Sul (PUCRS), Porto Alegre, Rio Grande do Sul, Brazil.; IV Undergraduate Student of Pharmacy, Universidade Federal de Ciências da Saúde de Porto Alegre (UFCSPA), Porto Alegre, Rio Grande do Sul, Brazil.; V MD. Clinical Geneticist, Universidade Federal de Ciências da Saúde de Porto Alegre (UFCSPA) and Complexo Hospitalar Santa Casa de Porto Alegre (CHSCPA), Porto Alegre, Rio Grande do Sul, Brazil.; VI PhD. Professor of Clinical Genetics and of the Postgraduate Program in Pathology, Universidade Federal de Ciências da Saúde de Porto Alegre (UFCSPA), and Clinical Geneticist, Universidade Federal de Ciências da Saúde de Porto Alegre (UFCSPA) and Complexo Hospitalar Santa Casa de Porto Alegre (CHSCPA), Porto Alegre, Rio Grande do Sul, Brazil.

**Keywords:** Goldenhar syndrome, Karyotype, In situ hybridization, fluorescence, Fanconi anemia, Mosaicism, Twinning, monozygotic, Síndrome de Goldenhar, Cariótipo, Hibridização in situ fluorescente, Anemia de Fanconi, Mosaicismo, Gemelaridade monozigótica

## Abstract

**CONTEXT AND OBJECTIVE:**

Oculo-auriculo-vertebral spectrum (OAVS) is considered to be a defect of embryogenesis involving structures originating from the first branchial arches. Our objective was to describe the clinical and cytogenetic findings from a sample of patients with the phenotype of OAVS.

**DESIGN AND SETTING:**

Cross-sectional study in a referral hospital in southern Brazil.

**METHODS:**

The sample consisted of 23 patients who presented clinical findings in at least two of these four areas: orocraniofacial, ocular, auricular and vertebral. The patients underwent a clinical protocol and cytogenetic evaluation through high-resolution karyotyping, fluorescence *in situ* hybridization for 5p and 22q11 microdeletions and investigation of chromosomal instability for Fanconi anemia.

**RESULTS:**

Cytogenetic abnormalities were observed in three cases (13%) and consisted of: 47,XX,+mar; mos 47,XX,+mar/46,XX; and 46,XX,t(6;10)(q13; q24). We observed cases of OAVS with histories of gestational exposition to fluoxetine, retinoic acid and crack. One of our patients was a discordant monozygotic twin who had shown asymmetrical growth restriction during pregnancy. Our patients with OAVS were characterized by a broad clinical spectrum and some presented atypical findings such as lower-limb reduction defect and a tumor in the right arm, suggestive of hemangioma/lymphangioma.

**CONCLUSIONS:**

We found a wide range of clinical characteristics among the patients with OAVS. Different chromosomal abnormalities and gestational expositions were also observed. Thus, our findings highlight the heterogeneity of the etiology of OAVS and the importance of these factors in the clinical and cytogenetic evaluation of these patients.

## RESUMO

**CONTEXTO E OBJETIVO:**

O espectro oculoauriculovertebral (EOAV) é considerado um defeito de embriogênese envolvendo estruturas originadas a partir dos primeiros arcos branquiais. Nosso objetivo foi descrever os achados clínicos e citogenéticos de uma amostra de pacientes com fenótipo de EOAV.

**TIPO DE ESTUDO E LOCAL:**

Estudo transversal em um hospital de referência no sul do Brasil.

**MÉTODOS:**

A amostra foi composta de 23 pacientes que apresentaram achados clínicos em pelo menos duas das quatro áreas: orocraniofacial, oculares, auriculares e vertebrais. Os pacientes foram submetidos a um protocolo clínico e avaliação citogenética através do cariótipo de alta resolução, hibridização *in situ* fluorescente para as microdeleções 5p e 22q11 e pesquisa de instabilidade cromossômica para anemia de Fanconi.

**RESULTADOS:**

Alterações citogenéticas foram observadas em três casos (13%) e consistiam de: 47,XX,+mar; mos 47,XX,+mar/46,XX e 46,XX,t(6;10)(q13;q24). Observamos casos de EOAV com história de exposição gestacional à fluoxetina, ácido retinoico e *crack*. Um dos nossos pacientes foi um gêmeo monozigótico discordante que teve restrição de crescimento assimétrica durante a gravidez. Nossos pacientes com EOAV foram caracterizados por um amplo espectro clínico e alguns apresentaram achados clínicos atípicos como um defeito de redução de membro inferior e um tumor do braço direito, sugestivo de hemangioma/linfangioma.

**CONCLUSÕES:**

Verificamos grande variedade de características clínicas entre os pacientes com EOAV. Também foram observadas diferentes anomalias cromossômicas e exposições gestacionais. Assim, nossos achados salientam a heterogeneidade da etiologia do EOAV e a importância desses fatores na avaliação clínica e citogenética desses pacientes.

## INTRODUCTION

Oculo-auriculo-vertebral spectrum (OAVS) or Goldenhar syndrome is a condition with a great variety of clinical manifestations and its frequency ranges from 1:4,000 to 1:45,000 live births.^,^^2^ Its main findings consist of abnormalities, usually asymmetrical, involving the face, eyes, ears and spine. OAVS is considered to be a defect of embryogenesis involving structures originating from the first branchial arches, possibly due to vascular injury^1^ or altered migration of neural crest cells.^3^ Although individuals affected by OAVS are cytogenetically normal, similar phenotypes can be found in patients with different chromosomal^4^ and gene abnormalities,^3^^,^^5^ as well as those exposed to some teratogens.^3^ Thus, it is important to make a differential diagnosis. Conditions that may present similar features and thus are considered to be important differential diagnoses with OAVS include branchio-oto-renal syndrome, Townes-Brocks syndrome and the VACTERL association [vertebral defects (V), anal atresia (A), cardiac malformations (C), tracheoesophageal fistula with esophageal atresia (TE), renal dysplasia (R) and limb anomalies (L)].^3^

Few prospective studies have used the same clinical criteria for patient selection and have systematically performed cytogenetic evaluation through fluorescent *in situ* hybridization (FISH) in all individuals with OAVS.^6^^,^^7^

## OBJECTIVE

The aims of our study were to describe the clinical and cytogenetic features of a sample of patients with the phenotype of OAVS and to evaluate family factors and pregnancy outcomes such as exposure to teratogenic agents and maternal diseases that may be related to the etiology of OAVS.

## METHODS

Our sample was composed of patients with the phenotype of OAVS attended in the Department of Clinical Genetics of Universidade Federal de Ciências da Saúde de Porto Alegre (UFCSPA), Complexo Hospitalar Santa Casa de Porto Alegre (CHSCPA). The inclusion criteria used were those suggested by Strömland et al.,^8^ i.e. patients who presented the phenotype of OAVS with clinical abnormalities in at least two of the following areas: 1) orocraniofacial, 2) ocular, 3) auricular and 4) vertebral.

The patients were identified through their registrations at the clinic and were recruited through mail, phone or direct contact during the medical consultation. From a total of 46 patients evaluated from 1975 to 2012, 23 were located and agreed to participate. Some of them have already been described retrospectively in three studies that evaluated the frequency of abnormalities of the central nervous system, cardiac malformations and ear abnormalities among patients with OAVS.^9^^,^^10^^,^^11^ These studies were performed in the same institution and covered the period from 1975 to 2007.

For each patient, an evaluation form seeking identity, medical history, pregnancy and family data, physical/dysmorphological findings and results from complementary tests was completed. Abnormalities in other organs or systems (such as the results from ophthalmic, otorhinolaryngological, cardiac, neurological and radiological evaluations) were also noted.

All of them underwent high-resolution GTG-banding karyotyping in peripheral blood with scoring of 100 metaphase plaques in order to increase the possibility of detecting chromosomal mosaicism. The fluorescence in situ hybridization (FISH) technique for 22q11 and 5p microdeletions, using the Tel Vysion TM TUPLE 1 and Tel Vysion TM 5p SG probes (Abbott Molecular Inc., Des Plaines, Illinois, United States), was performed in accordance with the protocol provided by the probe manufacturer. We also conducted a search for chromosomal instability using diepoxybutane, in accordance with the protocol suggested by Auerbach.^12^ This method is used to identify patients with Fanconi anemia.

This study was approved by our institution’s Ethics Committee and all patients or guardians signed an informed consent form.

## RESULTS

Out of the total of 23 patients who fulfilled the clinical criteria for inclusion in the study, three showed abnormalities in their karyotypes: 47,XX,+mar (patient 22); mos47,XX,+mar/46,XX (patient 23) and 46,XX,t(6;10)(q13;q24) (patient 21). In one case, constitutional polymorphism was observed, consisting of 9qh inversion: 46,XX,inv(9)(p11q13) (patient 16). No cases of 22q11 and 5p microdeletions were found using FISH, or Fanconi anemia using studies on chromosomal breaks. However, it was noteworthy that the marker chromosome of the patient without mosaicism (patient 22) was also marked by the TUPLE 1 probe, thus indicating that at least part of it originated from chromosome 22. No karyotyping studies were performed on the parents.

Among the patients with normal cytogenetic analyses, i.e. those who fulfilled all the criteria used for diagnosing OAVS, their ages ranged from 2 days to 24 years, with an average of 6.7 years and a median of 7 years. Male patients predominated (60%), and most of the sample (55%) fulfilled three of the criteria of Strömland et al.^8^ (Table 1). The average maternal age at the child birth was 29.2 years and the mean paternal age was 33.2 years. Regarding diseases or complications among the patients’ mothers, these were observed in nine cases (45%): three mothers with vaginal bleeding in the first trimester; two with hypertension; one with insulin-dependent diabetes who presented ketoacidosis during pregnancy (patient 18); two with gestational diabetes; one with depression, who had made use of fluoxetine (patient 4) and one who had used of retinoic acid in the first trimester (patient 17). Four of the patients’ mothers were smokers, two reported alcohol intake and one reported use of crack (patient 3).

**Table 1.**
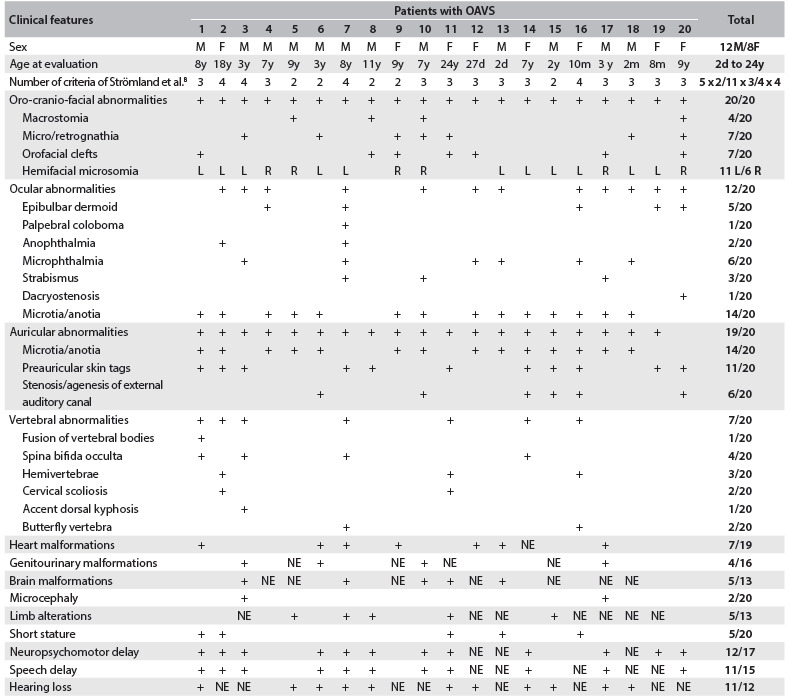
Clinical characteristics of patients with oculoauriculovertebral spectrum (OAVS) in our sample

M = male; F = female; d = days; m = months; y = years; L = left; R = right; NE = not evaluated.

Obstetric ultrasound examination had been done in 14 cases (70%), and abnormalities were identified in only 50%. Ultrasound revealed polyhydramnios in two cases; oligohydramnios and cleft lip in one; congenital heart disease in two; and intrauterine growth restriction in two.

**Figure 1.**
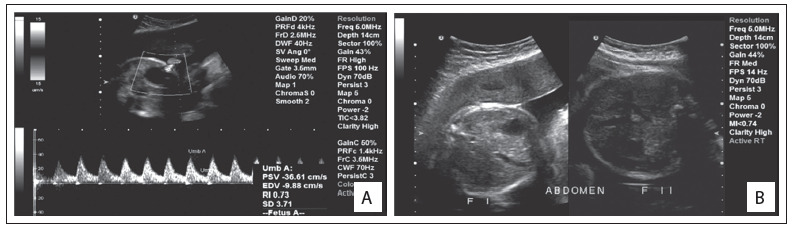
Obstetric ultrasound showing increased resistance of umbilical artery of fetus I (with oculo-auriculo-vertebral spectrum) and difference in abdominal circumference between fetus I (abnormal) and fetus II (normal).

**Figure 2.**
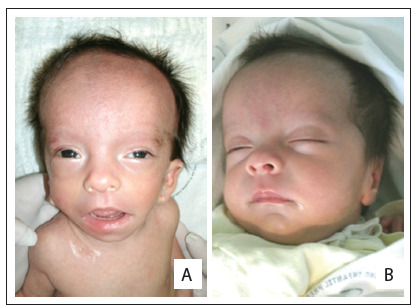
Craniofacial aspect of the twins. The patient with the phenotype of oculo-auriculo-vertebral spectrum can be seen on the left (permission was obtained from the patients’ parents for presentation).

**Figure 3.**
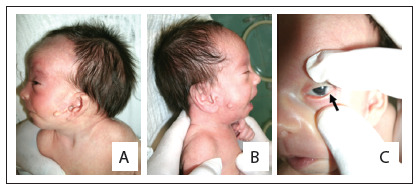
Craniofacial appearance of the twin with oculo-auriculo-vertebral spectrum. Note especially the preauricular skin tags, low-set ears (A and B) and epibulbar dermoid (C).

**Figure 4.**
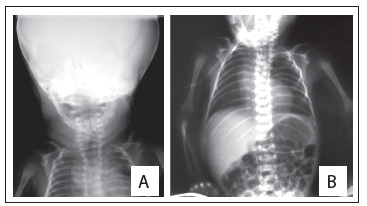
Radiographs of the oculo-auriculo-vertebral spectrum twin showing hypoplasia of the left mandible, and vertebral and rib abnormalities (including hemivertebrae and butterfly vertebrae) (A and B).

One of these cases consisted of a discordant monozygotic twin, i.e. the other twin was normal. There was an asymmetric growth restriction, affecting only the fetus with OAVS (twin 1) (Figure 1). Assisted reproduction techniques were not used in this case. There were no signs of fetal transfusion, oligohydramnios or polyhydramnios. The sizes of bladders were also appropriate for the gestational age. Increased resistance of the fetal umbilical artery was observed in the fetus with OAVS (this was 0.73, while the normal range is from 0.46 to 0.7), without signs of fetal centralization. The biophysical profile was also lower in twin 1, due to the absence of breathing movements. These children were born weighing 1,530 g (twin with OAVS) and 2,100 g (normal twin) (Figures 2, 3 and 4).

There was no consanguinity between the parents of the patients with OAVS in our sample. Three cases showed a pattern suggestive of familial recurrence, with preauricular tags in the maternal grandfather of case 8, grade IV microtia and ear canal agenesis in the father of case 15, and hemifacial microsomia in the father of case 18.

The main features presented by the patients with normal cytogenetic analysis and OAVS can be seen in Table 1. Seven cases (35%) had congenital heart disease; eleven cases (55%) had neuropsychomotor delay, ten cases had speech delay and eight cases had learning difficulties. Of these, some underwent further central nervous system investigation (nine patients underwent brain computed tomography; two, magnetic resonance imaging; and three, both examinations), and abnormalities were observed in 40% of these patients. Eight patients underwent computed tomography of the ear and mastoid, and abnormalities were found in seven of them (87.5%). Radiographs of the spine were performed on eleven patients, of whom seven (63%) had vertebral malformations. None of them had any radial abnormality. Sixteen patients underwent abdominal ultrasound, and alterations were identified in 31% of these patients (Table 1).

## DISCUSSION

Despite all the efforts to elucidate the genetic cause of OAVS and the existence of numerous candidate genes, the cause has not yet been found and the impression that persists is that this condition has complex etiology.^13^ This reinforces the hypothesis that genetic heterogeneity and a variety of pathogenic mechanisms are contributory causes of this clinical entity, including epigenetic inheritance.^14^

Currently, there is no specific diagnostic test for OAVS. Therefore, clinical assessment remains the first tool to be used, in association with cytogenetic and imaging tests. It has been reported that the clinical manifestations of OAVS overlap with those of other entities, such as the VACTERL,^15^ CHARGE and OEIS associations.^16^ According to some authors, the VACTERL association and OAVS are a spectrum of anomalies derived from mesenchymal structures, probably caused by the same pathogenic mechanism.^15^ The role of exogenous factors at different periods of embryogenesis triggers a similar pattern of malformations, but this pattern appears to be more related to the time of disruption than to the pathogen itself.^15^ These reports reinforce the need for further investigation of the various systems using imaging, so that the diagnosis can be established more consistently.

Similarly, the VACTERL association may also have clinical findings that overlap with Fanconi anemia, such as radial, cardiac and renal abnormalities.^17^ In the light of the genetic predisposition towards cancer among individuals with Fanconi anemia, some authors have recommended that a differential diagnosis should be established, which can be accomplished by testing for chromosome breakage using an inductor, commonly diepoxybutane.^17^^,^^18^ It can thus be noted that the clinical manifestations of OAVS may overlap with both the VACTERL association and Fanconi anemia. Therefore, we also searched for Fanconi anemia in our study. Although we did not find any patient with Fanconi anemia, our study is the first in the literature to perform such analysis. Interestingly, Vendramini et al.^19^ reported 14 cases with the phenotype of OAVS that also presented radial defects, which is a finding often seen in Fanconi anemia,^18^ and proposed that this would represent a subset within OAVS. However, no analysis was performed to rule out Fanconi anemia in these cases. Therefore, we suggest that this evaluation should be made in further samples of patients with the phenotype of OAVS, in order to verify or rule out the existence of such an association, since most cases of OAVS do not present any definite etiology. This would be of great importance for diagnostic definition and appropriate management, and for genetic counseling of patients.

In our sample, one patient with the phenotype of OAVS had 47,XX,+ mar/46,XX mosaicism. Standardized analysis on a larger number of metaphases enabled identification of this abnormality. This individual presented a diagnosis of cat-eye syndrome and was also part of the study by Rosa et al.^20^ Mosaic abnormalities may be unilateral or bilateral, depending on the period of blastogenesis during which the mutation occurs. The earlier the event occurs, the greater the individual’s impairment is. Typically, phenotypic mosaic alterations never occur in all tissues, and thus, many of them are asymmetrical, and several present associated malformations,^21^^,^^22^ as is observed in OAVS. Furthermore, it is noteworthy that mosaicism has been considered to be a possible cause of other conditions that are often associated with asymmetry and a possible cause of vascular disruptive, as in the Poland sequence.^22^^,^^23^ The observation, both in the literature and in our study, of cases with the phenotype of OAVS showing chromosome mosaicism^20^^,^^24^^,^^25^ emphasizes the etiologically heterogenous nature of OAVS and the importance of performing karyotype analysis with a large cell count. Since the proportions of altered cells are very variable, it seems appropriate to suggest that, in cases of individuals with the phenotype of OAVS, the metaphase analysis should be expanded to increase the possibility of detecting mosaicism. In our study, we analyzed a total of 100 metaphases for each patient. This analysis identifies mosaicism up to 3%, for a 95% confidence interval.^26^ It allowed us to identify one case of mosaicism, which was composed of a cell line with a marker chromosome present in 68 cells and a cell line with 32 normal cells (mos 47,XX,+mar [68]/46,XX [32]). The clinical evaluation on this patient was consistent with a diagnosis of cat-eye syndrome, i.e. the marker chromosome was inv dup(22)(pter->q11.2::q11.2->pter).^20^

Because phenotypes similar to OAVS have been described in patients with 22q11 and 5p microdeletions,^27^^,^^28^ we conducted a study on these microdeletions through FISH. However, we did not find any patients with these cytogenetic abnormalities. Thus, our results were similar to those of Tasse et al.^6^ and Digilio et al.^7^

As already described in the literature, we also observed cases of OAVS relating to drug or teratogenic exposure in pregnancy in our sample. These included one patient with gestational exposure to fluoxetine^29^ and another to retinoic acid.^30^ These reports reinforce the likelihood that the phenotype of OAVS is associated with these medications. Interestingly, in experimental models, associations with exposure to serotonin uptake inhibitors such as fluoxetine and development of craniofacial abnormalities have been described.^31^ Moreover, the occurrences of cases of OAVS in our study, in which the mothers had had vaginal bleeding during the first trimester of pregnancy, and had developed diabetes *mellitus*, reinforce the hypothesis of vascular disruption.^1^^,^^3^ In our sample, there was also a patient whose mother had been exposed to crack, an impure form of cocaine, and Lessick et al.^32^ reported a case of OAVS that was correlated with use of this illicit drug.

Today, it is known to be possible to make a prenatal diagnosis of OAVS through fetal ultrasound from the fourteenth week of pregnancy onwards, especially in cases with more severe manifestations.^33^ However, it is noteworthy that among the 14 patients in our study who underwent fetal sonographic evaluation, half of them had test results that were considered normal. For example, during the pregnancy of the mother of case 7, who had multiple malformations, several ultrasound examinations were conducted but they only revealed polyhydramnios.

In our sample, there was also a case of a twin with the phenotype of OAVS. This patient was briefly described by Goetze et al.^34^ She was a discordant monozygotic twin. As in other studies that have observed a large number of cases of OAVS between concordant or discordant twins,^35^ the impression that persists is that there is an association between twinning and OAVS. In such cases, the causes of OAVS may be inherent to the twinning, be of maternal origin or be related to both.^36^ Wieczorek et al.^35^ also found a correlation between OAVS and use of assisted reproductive techniques. These findings are concordant with the concept of overripeness ovopathy.^35^ It was noteworthy in our case, however, that there was possibly a vascular component relating to OAVS, since only the affected fetus showed increased resistance in the umbilical artery and asymmetrical growth restriction. As previously reported, a hypothesis of vascular disruption has been etiologically correlated with OAVS.^1^^,^^3^

With regard to family history, consanguinity between parents has rarely been reported, as also observed in our sample.^37^ We observed that three patients (15%) had family members, especially first-degree kin, with findings pertaining to OAVS, thus suggesting a possible pattern of familial recurrence. Rollnick and Kaye^38^ found that 45% of the patients with OAVS in their sample had a similar story. They especially had minor anomalies, such as preauricular tags. Tasse et al.^6^ postulated that more severe malformations may appear in the offspring of patients who have these minor ear anomalies and proposed that they should be included as minor criteria of the spectrum.

**Figure 5.**
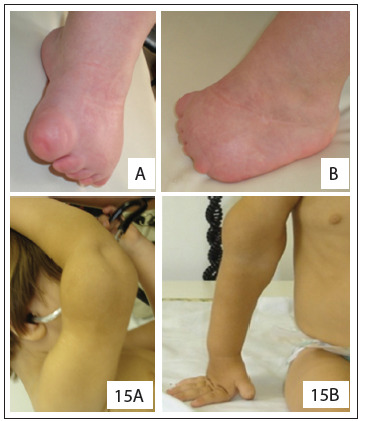
Details of the patients presenting atypical findings. Note lower-limb reduction defect (patient 5) and tumor in the distal portion of the right arm, suggestive of a hemangioma/lymphangioma (patient 15) (permission was obtained from the patients’ parents for presentation).

As noted in the results, patients with OAVS are characterized by a wide clinical spectrum. Thus, these patients usually require multidisciplinary assessment and monitoring, especially early in life, in order to optimize treatment. The severity of the condition is also variable, and cardiac malformations are common. Some patients may also present OAVS with atypical findings, perhaps reflecting the heterogeneity associated with its etiology. For example, our patient 5 showed a lower-limb reduction defect suggestive of vascular disruption. Interestingly, as seen before, this is considered to be one of the most accepted hypotheses for explaining OAVS.^1^^,^^3^ Our patient 15 had a tumor in the distal third of the right arm, suggestive of hemangioma/lymphangioma, a finding not previously described in the literature (Figure 5). None of our patients with OAVS had any radial abnormality of the upper limbs, which is a common finding in Fanconi anemia. Hence, absence of this abnormality may be related to the fact that we found no patients with Fanconi anemia in our sample.

Despite the difficulty of establishing the specific etiological diagnosis, we believe that this should always be pursued in patients presenting the phenotype of OAVS. Early recognition, even at the intrauterine stage, and detailed understanding of the issues relating to the etiology and clinical features of patients with OAVS are essential for its management and for genetic counseling. In cases of OAVS, the empirical risk of recurrence in future pregnancies of a couple with an affected child is considered to be about 2% to 3%.^3^

OAVS is considered to be a heterogeneous condition characterized by a wide clinical spectrum. It is important to be aware that some chromosomal abnormalities may present clinical features that can simulate the findings observed in OAVS. Because of this, cytogenetic evaluation of these patients, using different conventional and molecular cytogenetic techniques, is crucial for correct diagnosis and consequently for proper management and genetic counseling. Despite the absence of patients with Fanconi anemia, this study attempted to emphasize the importance of making a differential diagnosis between OAVS and this condition. Furthermore, we suggest that metaphase analysis through karyotype examination should be expanded in cases without a definite etiology, in order to identify possible mosaicism. In cases of OAVS, a detailed evaluation of family and gestational history, giving particular emphasis to possible exposures, is also very important. More studies with large samples are still needed so as to better understand the different factors and characteristics relating to the etiology of OAVS.

## CONCLUSIONS

In our study, we found a wide range of clinical characteristics among the patients with OAVS. Some of them were atypical, such as lower-limb reduction defect and a limb tumor suggestive of hemangioma/lymphangioma. These may provide clues for the etiology of OAVS. The cytogenetic evaluation performed through GTG-banding, the FISH technique for 22q11 and 5p microdeletions and investigation of chromosomal instability using diepoxybutane was able to identify three carriers of chromosomal abnormalities (13% of the sample), which highlights the importance of these evaluations among patients with OAVS. We observed cases of OAVS with a gestational history of exposure to fluoxetine, retinoic acid and crack, along with a discordant monozygotic twin. These factors have been correlated with the etiology of OAVS. Thus, our findings highlight the heterogeneity of the etiology of OAVS and the importance of these factors in the clinical and cytogenetic evaluation of these patients.
